# Dipole Orientation Engineering in Crosslinking Polymer Blends for High‐Temperature Energy Storage Applications

**DOI:** 10.1002/advs.202405730

**Published:** 2024-08-29

**Authors:** Zizhao Pan, Li Li, Fei Jin, Jiufeng Dong, Yujuan Niu, Liang Sun, Li Tan, Yuqi Liu, Qing Wang, Hong Wang

**Affiliations:** ^1^ Department of Materials Science and Engineering Southern University of Science and Technology Shenzhen Guangdong 518055 China; ^2^ Department of Materials Science and Engineering The Pennsylvania State University University Park Pennsylvania 16802 USA

**Keywords:** crosslinking networks, dipole orientation engineering, high energy density, high polarization, high‐temperature polymer dielectrics

## Abstract

Polymer dielectrics that perform efficiently under harsh electrification conditions are critical elements of advanced electronic and power systems. However, developing polymer dielectrics capable of reliably withstanding harsh temperatures and electric fields remains a fundamental challenge, requiring a delicate balance in dielectric constant (*K*), breakdown strength (*E*_b_), and thermal parameters. Here, amide crosslinking networks into cyano polymers is introduced, forming asymmetric dipole pairs with differing dipole moments. This strategy weakens the original electrostatic interactions between dipoles, thereby reducing the dipole orientation barriers of cyano groups, achieving dipole activation while suppressing polarization losses. The resulting styrene‐acrylonitrile/crosslinking styrene‐maleic anhydride (SAN/CSMA) blends exhibit a *K* of 4.35 and an *E*_b_ of 670 MV m^−1^ simultaneously at 120 °C, and ultrahigh discharged energy densities (*U*_e_) with 90% efficiency of 8.6 and 7.4 J cm^−3^ at 120 and 150 °C are achieved, respectively, more than ten times that of the original dielectric at the same conditions. The SAN/CSMA blends show excellent cyclic stability in harsh conditions. Combining the results with SAN/CSMA and ABS (acrylonitrile‐butadiene‐styrene copolymer)/CSMA blends, it is demonstrated that this novel strategy can meet the demands of high‐performing dielectric polymers at elevated temperatures.

## Graphical abstract

Scalable high‐*K*, high‐breakdown, low‐loss cross‐linked polymer blends induced by asymmetric dipole pairs with different dipole moments achieve ultra‐high discharge energy density at high temperatures. This work fills an important gap in the design of high‐temperature, high‐*K* materials, representing a breakthrough in the development of high‐energy‐density polymer dielectrics.

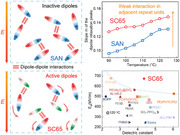

## 1 Introduction

Polymer dielectrics have been widely used in power grids, photovoltaic wind power, and pulse systems because of their high power density and excellent stability.^[^ ^1^, ^2^, ^3^, ^4^ ^]^ Polymers represented by biaxially oriented polypropylene (BOPP) have inherent advantages of low cost, easy processing, lightweight, high breakdown strength (*E*_b_), and excellent failure mechanism.^[^ ^5^, ^6^, ^7^ ^]^ However, as the temperature rises to 85 °C, BOPP exhibits a sharp deterioration in capacitive performance and working life. The relatively low dielectric constant (*K*) of BOPP (≈2.2) limits its discharged energy density (*U*_e_) and further hinders its application in high‐temperature capacitors.^[^ ^8^ ^]^ Many advanced electronic systems, such as electric vehicles, underground oil and gas exploration, and aerospace systems, urgently need polymer dielectrics that can function properly at higher temperatures (i.e., 150 °C) and high electric fields.^[^ ^9^, ^10^, ^11^ ^]^ Therefore, high *U*_e_ with high operating temperature is vital for the field of polymer dielectric applications.

The *U*_e_ of the dielectric materials is mainly determined by both the *K* and *E*_b_.^[^ ^4^, ^12^ ^]^ However, there is a negative correlation between *K* and *E*_b_, which can be expressed in terms of an empirical relationship as *E*_b_ ∝ *K* ^−0.65^.^[^ ^13^ ^]^ What's more, at elevated temperatures, the consequent softening of polymer chains and thermally excited charge carriers inhibited *U*_e_ due to the deterioration of *E*_b_ and charge‐discharge efficiency (*η*), suppressing their high‐temperature dielectric applications.^[^ ^14^, ^15^ ^]^ In the pursuit of high‐*K* dielectric polymers, the common routine is mainly composed of contributions from electron and dipole polarization.^[^ ^12^, ^16^ ^]^ For example, conjugated aromatic structures improve the *K* of fluorene polyester (FPE), polyimide (PI), polystyrene (PS), and polyether ether ketone (PEEK) due to the contribution of electronic polarization. But this strategy also leads to a significant decrease in their bandgap, resulting in deterioration of *E*_b_ and *η*, such as 57.8% for FPE and 18.2% for PI at 150 °C and 300 MV m^−1^.^[^ ^15^ ^]^ Although recent efforts including nanocomposites or nano coating modifications are encouraging for concurrently enhancing *K* and *E*_b_ at elevated temperatures,^[^ ^14^, ^17^, ^18^, ^19^, ^20^ ^]^ they are generally difficult for industrial‐scale production due to requirements of materials cost or laborious multi‐step synthesis and processing.

High dipole moment groups (─SO_2_‐ and ─CN, ≈4 Debye) are found to enhance dipole polarization of polymers ^[^ ^12^, ^21^, ^22^, ^23^ ^]^, resulting in significant improvements in *K*. With the contribution of CN and CO dipoles, cyanoethyl cellulose^[^ ^21^ ^]^ exhibited a *K* of 16.5 at room temperature. Sulfonylated poly(2,6‐dimethyl‐1,4‐phenylene oxide) (SO_2_‐PPO) displayed a higher *K* than PPO (3.1) at room temperature,^[^ ^12^ ^],^ e.g., 5.9 for SO_2_‐PPO_25_ (PPO containing 52% SO_2_ groups), 8.2 for SO_2_‐PPO_52_. Although the *K* of SO_2_‐PPO_52_ is higher, the rapid deterioration of *E*_b_ and *η* results in a lower *U*_e_ than SO_2_‐PPO_25_. The *U*_e_ of SO_2_‐PPO_25_ (≥90% *η*) sharply decreased from 18 J cm^−3^ at room temperature to only 2.1 J cm^−3^ at 150 °C. Clearly, the simple accumulation of polar groups in polymers cannot meet the requirements of high‐temperature and high‐electric‐field applications due to the substantial increase in loss caused by electrostatic interaction between dipoles and steric hindrance of adjacent groups. Pursuing enhanced *U*_e_ at high temperatures, the activity of dipoles should be rationally adjusted within heat‐resistant polymer structures to maximize dipolar polarization while simultaneously minimizing polarization loss.^[^ ^24^ ^]^

**Figure 1:**
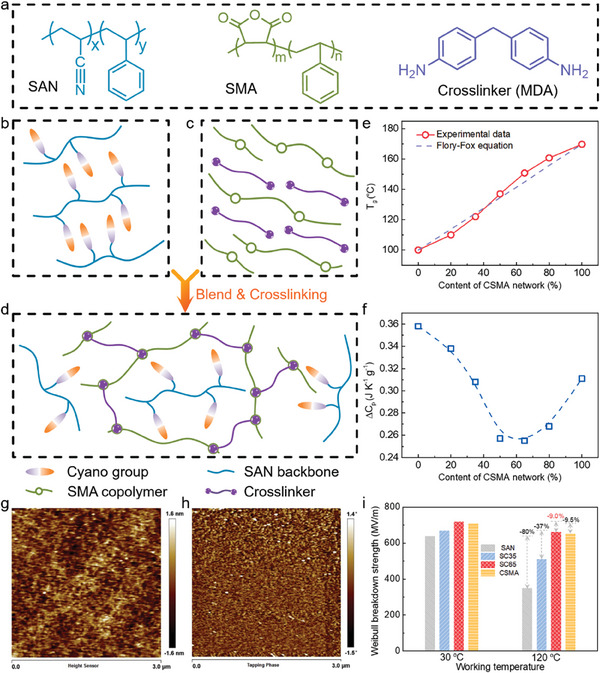
Structural design and characterization. a) Molecular formula of materials for structural design schematic of polymers and blends of b) SAN, c) CSMA, and d) the polymer blends. e) Glass transition temperature (*T_g_*) and f) change in specific heat capacity during glass transition (*ΔC_p_*) for the polymer blends at various CSMA content. Tapping‐mode AFM results for SC65 blends, topography g) and phase h) data with a scanning area of 3 µm × 3 µm; i) comparison of room‐temperature and high‐temperature *E*_b_ of blend polymer at various CSMA content.

The crosslinking structures can effectively suppress the movement of molecular chains at a broad range of temperatures without introducing conjugate structures. In addition, those trap centers of charges induced by crosslinking^[^ ^25^, ^26^ ^]^ are beneficial for improving the high‐temperature *U*_e_ and *η* of polymers. Therefore, we selected polar cross‐linked networks (dipole moment, 2.14 D) and polar polymers (4.0 D) to construct asymmetric dipole pairs with different dipole moments, weakening dipole–dipole interactions to active dipole orientation, endowing dipoles with stable orientation ability over a wide temperature range. As shown in **Figure** **1**a–d, SMA (styrene‐maleic anhydride copolymer) and SAN (styrene‐acrylonitrile copolymer), molecular compositions can be seen in Figure S1, Supporting Information) with the same segments are chosen for engineering interchain interactions and subsequent crosslinking in polymer blends. The design of copolymerized styrene groups ensures good compatibility with these two polymers. This strategy enhanced polymer blends’ *K* and *U*_e_ without sacrificing *E*_b_ and *η*, especially with high capacitive performance at high temperatures. The developed CSMA (crosslinking SMA) and SAN crosslinking blends represent a high *U*_e_ of 8.6 J cm^−3^ at 120 °C and 7.4 J cm^−3^ at 150 °C (≥90% *η*), exceeding the capacitive performance of the advanced dielectric polymers and nanocomposites at the same conditions.

## 2 Results and Discussion

### 2.1 Preparation and Characterization

Polymer blends consisted of SAN and SMA and then crosslinked by 4,4‐diaminodiphenylmethane (MDA, specific structures are shown in Figure S1, Supporting Information). Based on the content of CSMA (*φ*CSMA) in SAN/CSMA blends, the SAN/CSMA blends are abbreviated as SC, i.e., 65% CSMA in blends labeled as SC65. From Figure 1e and Figure S2 (Supporting Information), it can be seen that blends of different proportions have clear glass transition temperature (*T*_g_) peaks and only one transition point, indicating that they are well dispersed and uniformly mixed without phase separation.^[^ ^27^, ^28^ ^]^ Glass transitions of blends rise with the increase of *φ*CSMA, resulting from the crosslinked structure that inhibits the movement of the polymer chain. As a result, the crosslinked structure brings significant enhancement of the thermal stability in those polymer blends. As *φ*CSMA rises to 65%, the *T*_g_ of blends exceeds the fitting result of the Flory‐Fox equation, which may be caused by the deviation of effective crosslinking degree with the increasing amount of total maleic anhydride.^[^ ^26^ ^]^ The change of specific heat capacity (Δ*C*_p_) during the glass transition is decreased at first and then increases with *φ*CSMA, reaching its lowest value at the *φ*CSMA ≈65% (Figure 1f). The main reason for the change in specific heat during the polymer glass transition is the change in degrees of freedom and conformation as the polymer transitions from a highly self‐entangled state to a main‐chain extended state, as demonstrated in atactic polystyrene.^[^ ^28^, ^29^ ^]^ When the polarity of the blend formed by the thorough mixing of two polymers is most compatible with the solvent, a minimum value will be obtained. Therefore, the smallest Δ*C*_p_ in the *φ*CSMA = 0.65 indicates that the molecular chains of the two polymers are fully extended, which is conducive to optimizing the polymer structure and reducing defects.^[^ ^27^, ^28^ ^]^ It also reduces the concentration of cyanide groups, weakens the interaction between strong dipoles, and thus improves their dipole orientation. Figure 1g,h shows that the homogeneity of SC65 copolymer was evaluated by studying the phase shift in tapping‐mode atomic force microscope (AFM), revealing homogeneously distributed polymer blends, as compared with the pristine polymers (Figure S3, Supporting Information).

We then characterized the *E*_b_ of SAN/CSMA blend films between room temperature and 120 °C (Figure S4, Supporting Information), by using a two‐parameter Weibull distribution function, written as:^[^ ^33^ ^]^

(1) $$P\left(E\right)=1-\exp{\left(\frac{E}{E_{b}}\right)}^{\beta }$$

where *P* is the probability of breakdown failure, *E* is the experimentally measured electric breakdown field, *E*_b_ is the Weibull breakdown strength at 63.2% probability of breakdown, *β* is a shape parameter for evaluating the data scatter. As shown in Figure 1i, the SC65 blend film has the best *E*_b_ at both room temperature and 120 °C, higher than those of the individual polymer components. Even at 120 °C, SC65 showed a high *E_b_* (671 MV m^−1^), and the *E*_b_ receives only a 9% loss compared to that at room temperature. This may be due to the compact arrangement of the polymer chains, reducing the void and free volume and the point defects in the blends. Moreover, the crosslinked structure enhances Young's modulus of the materials (Figure S5, Supporting Information), effectively reducing the probability of dielectric breakdown under a high electric field.

### 2.2 Dielectric Properties and Capacitive Performance

**Figure 2:**
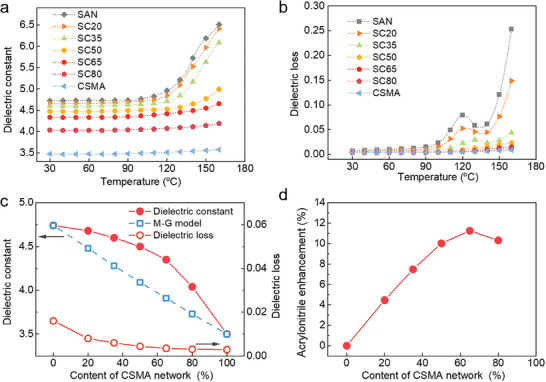
Dielectric properties. Temperature‐dependent a) *K* and b) dielectric loss of the polymer blends with various CSMA content at 1 kHz. c) Comparison of dielectric properties of the blended polymers at 30 °C and their fitting results of the Maxwell‐Garnett model. d) Dipole enhanced *η* of *K* in blended polymers with various CSMA content.

Variations of *K* and dielectric loss with temperature and frequency are studied, as shown in **Figure** **2**a,b, Figure S6 (Supporting Information). *K* values of those polymer blends are between 3.5 of CSMA and 4.75 of SAN at 1 kHz, positively correlated with the content of SAN. The crosslinked structure slightly reduces the *K* values of the polymer blends, but significantly optimizes their dielectric stability at elevated temperatures. The same trend is also reflected in dielectric loss (Figure 2b). The dielectric loss of SC blends is greatly suppressed with massive crosslinking networks, i.e., 0.016, 0.0035, 0.0031, and 0.003 for SAN, SC65, SC80, and CSMA, respectively, measured at 1 kHz and room temperature. Compared with CSMA, the *K* value of SC65 is enhanced from 3.5 to 4.35, exhibiting effective improvement in the *K* of polymer material with almost no introduction of dielectric loss. It can be found that the relaxation peak of dielectric loss in SAN is near 120 °C, exceeding its *T*_g_ by ≈20 °C. At this temperature, the relaxation chains of SAN significantly enhance the motion of dipoles, but the irregular motion of polymer segments also increases, resulting in a significant increase in *K* and dielectric loss. The relaxation peaks are also observed in SC20 and SC35, and the corresponding temperature of the transition peak increases. The relaxation peak disappears until the content of the crosslinked structure reaches 50%, and the dielectric loss decreases significantly. These results show that the introduction of the crosslinked network can reduce the dielectric loss of the SAN backbone and cyano groups. Maxwell‐Garnett model fitting shows that the *K* of the blends is obviously higher than the fitting result, indicating an extra contribution in blends that amplifies the *K* values of the corresponding polymer (Figure 2c). As the proportion of the crosslinked network rises, the contribution of cyano groups for total polarization also increases, as shown in Figure 2d. This may be due to the rigid structure of the crosslinked network that helps to provide the space required for local rotation of the cyano groups in the blends at a broad temperature range, thereby improving its dipole orientation ability and ultimately resulting in an additional enhancement of *K* (Figure S7, Supporting Information). The SC65 demonstrated a high‐temperature *E*_b_ of 670 MV m^−1^ and a *K* of 4.35, indicating that this crosslink‐blend strategy is conducive to breaking the limitation of the empirically inverse relation of *K*‐*E*_b_ without introducing additional dielectric loss. Therefore, SC blends have great potential for applications involving high‐temperature energy storage.

**Figure 3:**
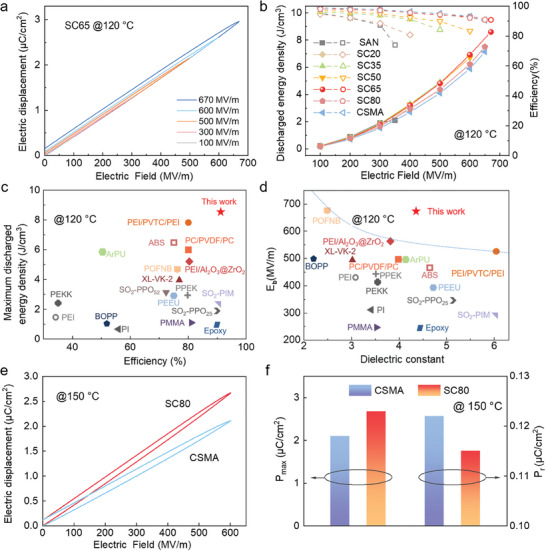
Capacitive performance. a) *D–E* loops of SC65 film at 120 °C. b) The *U*_e_ and *η* versus electric field of the blended polymers at 120 °C. c) Comparison of *U*_e_ at high temperatures between this work and other reported dielectric polymers and polymer composites at 120 °C. d) Comparison of *E*_b_ and *K* at high temperatures between this work and other reported dielectric polymers and polymer composites. SC65 shows a high *E*_b_ of 670MV m^−1^ and a *K* of 4.35 simultaneously at 120 °C. e) *D–E* loops and f) polarization values of the SC80 and CSMA films at 150 °C.

A unipolar electric displacement‐electric field (*D–E*) loop is performed to verify the performance of SAN and its crosslinked blends (Figure S8, Supporting Information). As shown in **Figure** **3**a, the SC65 displayed excellent energy storage performance at high temperatures, significantly higher than other components. It is worth noting that the SC65 with a high *K* can withstand 670MV m^−1^ electric field at 120 °C, resulting in the highest *U*_e_ of 8.6 J cm^−3^ with an *η* above 90% (Figure 3b), which also exceeds the current advanced high‐temperature polymers and composites (Figure 3c,d).^[^ ^12^, ^14^, ^16^, ^17^, ^25^, ^30^, ^31^, ^32^, ^33^, ^34^, ^35^, ^36^, ^37^ ^]^ As shown in Figure S9 (Supporting Information), the effective polarization (*P*_max_–*P*_r_) of SC65 is higher than that of other components due to the high polarization and low residual polarization of SC65 combines the positive characteristics of SAN and CSMA. The high *η* is attributed to the introduced crosslinked structure, which produces deep trapping centers to inhibit the internal charge movement.^[^ ^25^, ^26^ ^]^ It should be noted that its high energy storage performance also benefits from its high *K* and high *E*_b_. Thus, the dielectric performances of SC 65, like *K*, *E_b_*, *U_e_*, and resistance were superior to the two single‐component polymers (Figures S10 and S11, Supporting Information). As shown in Figure 3e and Figure S12 (Supporting Information), SC80 displayed an excellent performance at 150 °C, reaching 7.4 J cm^−3^ with 90% *η* at the electric field of 600 MV m^−1^, and exceeding a majority of advanced dielectric polymers and nanocomposites (Figure S13, Supporting Information).^[^ ^8^, ^10^, ^11^, ^19^, ^20^, ^25^, ^38^, ^39^, ^40^, ^41^, ^42^, ^43^, ^44^, ^45^, ^46^, ^47^ ^]^ SC80 blend displays a higher *P*_max_ (maximum polarization) and a lower *P*_r_ (remanent polarization) than CSMA (Figure 3f), simultaneously possessing high‐temperature resistance and high polarity, indicating the significant performance improvement and the superiority of the blended strategy.

**Figure 4:**
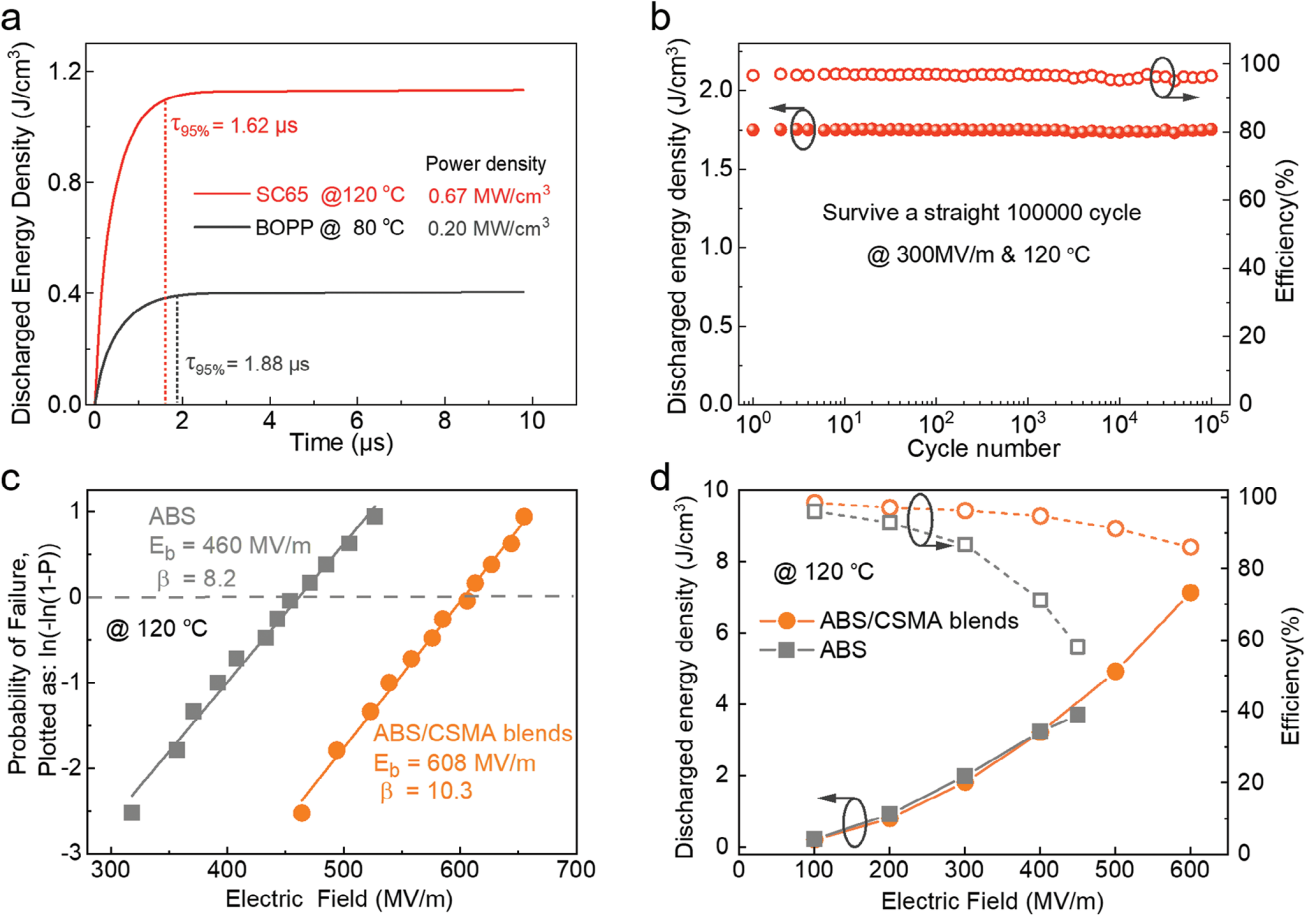
a) *U*_e_ and power density of BOPP and SC65 measured at high temperatures. b) Cyclic capacitive performance of SC65 with a high electric field of 300 MV m^−1^ at 120 °C. Comparison of c) *E*_b_ and d) *U*_e_ and *η* of ABS and ABS/CSMA blends at 120 °C.

Due to high *K* and low dielectric loss, SC65 exhibits excellent discharge power density, reaching 0.67 MV cm^−3^ at 200 MV at 120 °C, which is 3.35 times that of BOPP under the same electric field (**Figure** **4**a). In addition, the SC65 can maintain a very high *η* (>98%) for 100 000 charge and discharge cycles at 120 °C and 300 MV m^−1^, showing a stable cycle process and promising applications (Figure 4b). To verify the university of the interaction‐modulated dielectric enhancement, we further selected ABS since it also contains a styrene structure and dipole groups (Figure S14, Supporting Information). As is shown in Figure 4c, an enhanced *E*_b_ in ABS‐CSMA blends is revealed as compared with pristine ABS, exhibiting a significant improvement. The *D–E* loops test was also performed, as shown in Figure S15 (Supporting Information). Dielectric materials with higher *U*_e_ were found in ABS/CSMA blends, which demonstrated higher *U*_e_ and *η* at 120 °C (Figure 4d). This is similar to the optimization results of SAN polymer, signifying that the inter‐chain interaction engineering can be well applied to other polymers.

### 2.3 Dipole Orientation Engineering

**Figure 5:**
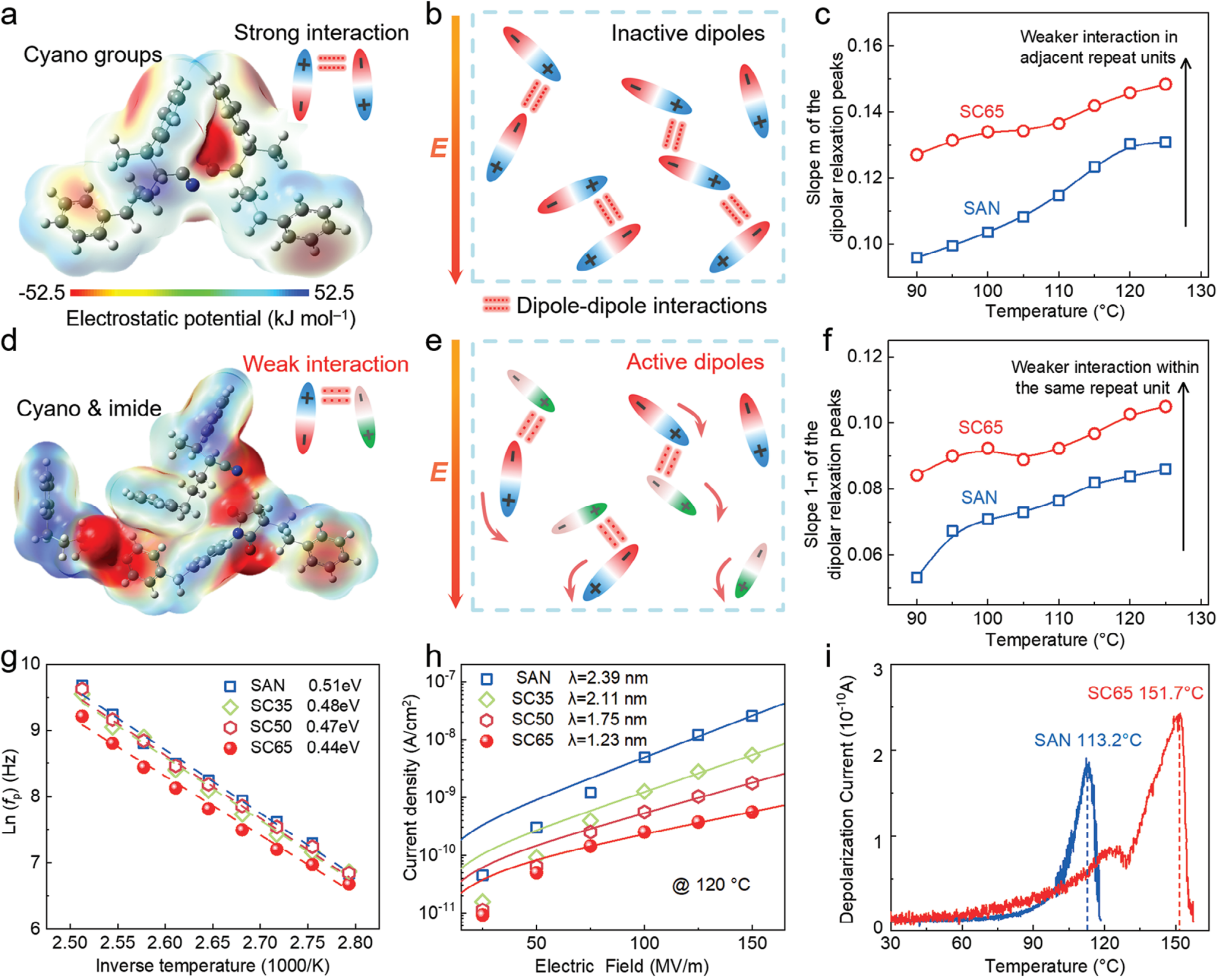
Backbone structures and electrostatic potential distribution of a) SAN&SAN and d) SAN&CSMA. The dipole‐dipole interactions in the b) SAN and e) SAN/CSMA blends. The slope of the relaxation peak for revealing interactions of polymer dipoles in c) adjacent units and f) the same repeat unit. g) Arrhenius function of a dipole‐reorientation potential barrier for the pristine SAN and the blends measured at different temperatures. h) The current density of SC blends and the pristine polymers with increasing electric field at 120 °C. The solid curves fit to hyperbolic sine. i) TSDC results of SAN and SC65.

By calculating the electrostatic potential of SAN, it is found that the dipole (cyano group) in the SAN presents a strong negative potential, which is in sharp contrast to the potential of the adjacent main chain vinyl structure (**Figure** **5**a). The strong negative and positive electrostatic potential of dipoles easily generate interaction forces, resulting in the coupling interactions in the polymer condensation process, which inhibit the orientation of dipoles at certain electric fields (Figure 5b). Accordingly, we improved the activity of dipoles by introducing lower‐polarity imide cross‐linking structures. The imide structures in CSMA exhibit significant positive electrostatic potentials and demonstrate a strong coupling tendency toward cyano groups (Figure 5d), forming asymmetric dipole pairs with different dipole moments. As shown in density functional theory (DFT) calculations (Figure 5a,d; Figure S16, supporting information), the interacting cross‐linking structures with cyano groups exhibit a high dipole moment (4.50 D), much stronger than the segments of SAN with dipole‐dipole interactions (0.68 D). It can be observed that the dipole‐dipole interactions of cyano groups greatly inhibit their polarization contribution. The interaction energy of CSMA‐SAN is −464.3 kJ mol^−1^, lower than SAN‐SAN (658.6 kJ mol^−1^), indicating that dipole activity in CSMA‐SAN is significantly improved than that in SAN‐SAN. Therefore, due to the construction of asymmetric dipole pairs and the weak interactions in CSMA‐SAN, the orientation capability of dipoles is expanded (Figure 5e), resulting in additional enhancement of *K* in the SC blends.

In the real relaxation process, the polymer group cannot undergo the relaxation process on its own without affecting the motion of the surrounding groups. There is a significant interaction between the microscopic particles (like polar groups) in the dielectric materials, which interact with each other through electrostatic forces, electro‐mechanical forces accompanied by changes in microstructure, chemical reactions, etc. The interaction between groups is further analyzed with the Dissado‐Hill Model,^[^ ^48^, ^49^ ^]^ which can focus on the coupled relaxation of adjacent groups. In the Dissado‐Hill model, the shape function of polarizability is,

(2)

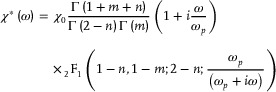

where, *Γ*() is the gamma function, _2_ *F*_1_(a, b; c; z) is a Gaussian hypergeometric function. The dielectric response process with relaxation peak can be fully described by four parameters, such as the amplitude of the polarizability *^χ^*_0_, the frequency of relaxation peak *ω*_p_, and the coefficient parameters *m* and *n*. *^χ^*_0_ reflects the concentration of dipoles during polarization. *ω*_p_ is the frequency corresponding to the maximum loss, exhibiting the frequency corresponding to the redirection of the polar groups. The parameters *n* and *m* depend on the slope of the dielectric spectrum at high and low frequencies.^[^ ^48^ ^]^ The smaller the interaction in adjacent polar groups and the same polar group unit, the closer the *m* and 1‐*n* values are to 1, respectively. They reflect the correlation coefficient of group motion and the interaction between groups.^[^ ^49^ ^]^ It can be observed that if *m* = 1 and *n* = 0, there is no interaction between the groups, and the Dissado‐Hill model can be simplified into the Debye model. With structural reinforcement of CSMA network, the rigid network in SC65 can effectively weaken the cyano dipole interaction (Figure 5c,f), and provide more favorable orientation space for cyano groups, reducing the activation energy required for orientation, and strengthening the cyano group movement. By monitoring the responses of polar groups at different temperatures and frequencies, the dipole‐reorientation potential barrier of SAN and SC blends is measured by fitting the dielectric loss (Figure 5g). Potential barriers of SC blends gradually decrease, reaching their lowest point at SC65. This reveals the enhanced orientation ability of dipoles with increasing content of the crosslinked structure, particularly under electric field conditions (Figure S17, Supporting Information), which is consistent with the DFT results. To establish a visualized configuration, we combined the designed molecular structures to create polymer and polymer blends (SAN, SC35, SC65, and SC80) as shown in Figure S18 (Supporting Information). Based on the variation of the total energy of cyano groups with torsion angle and the minimum total energy in SAN and SC blends (Figure S19, Supporting Information), we found that as the amount of CSMA increased, the cyano energy reached its highest at 65% and continued to decrease at 80%. This indicates that the cyano activity of SC65 is the highest among these polymers, making it easier to respond in an electric field environment and achieve a higher dielectric constant response. The leakage current density results also show that the crosslinked structure in blends is beneficial in reducing the leakage current density of SC blends (Figure 5h). The fitting curves show that the distance of hopping conduction in SC65 is the smallest (1.23 nm), indicating that the existence of deep traps in the internal crosslinked structure can effectively improve its *η*. The thermally stimulated depolarization current (TSDC) measurement proves that crosslinked structure produces extra carrier trap centers in blends with higher depolarization peak temperature and depolarization charge density (Figure 5i).

## 3 Conclusion

We propose a strategy aimed at modulating the oriented polarization of polymer dipoles by employing polar cross‐linked networks with asymmetric dipole moments. By weakening the dipole‐dipole interactions among adjacent dipoles, and reducing the potential barriers for dipole reorientation in polymer blends, we enhance the oriented polarization of polymer dipoles while reducing polarization losses. The specific crosslinking networks in the designed polar polymer blends balance significantly the electrical, and thermal properties of high‐performance polymer dielectrics, e.g., high dielectric constant, high breakdown strength, high glass transition temperatures, and low dielectric loss, achieving excellent energy storage densities of 8.6 J cm^−3^ (120 °C) and 7.4 J cm^−3^ (150 °C). An in‐depth exploration of the underlying mechanism in dipole optimization reveals a targeted regulation of dipole‐dipole interactions and a reduction in the potential barrier associated with dipole rotation. The dipole orientation engineering holds practical promises for high‐performance thin films, which can be extended to copolymers with similar structures and applied to high‐performance gate dielectric materials, providing a new way to optimize dielectric materials from a nanoscale.

## Conflict of Interest

The authors declare no conflict of interest.

## Supporting information

Supporting Information

## Data Availability

The data that support the findings of this study are available from the corresponding author upon reasonable request.
