# Supplementary material for: Dipole Orientation Engineering in Crosslinking Polymer Blends for High‐Temperature Energy Storage Applications
Source: Adv Sci (Weinh). 2024 Aug 29;11(40):2405730. doi: 10.1002/advs.202405730 (PMC11516106; doi:10.1002/advs.202405730)
Supplement: Supplementary file 1 — Supporting Information [file ADVS-11-2405730-s001.docx]

Electronic Supplementary Information

Asymmetric Dipoles in Crosslinking Polymer Blends for High-Temperature Energy Storage Applications

*Zizhao Pan, Li Li, Fei Jin, Jiufeng Dong, Yujuan Niu, Liang Sun, Li Tan, Yuqi Liu, Qing Wang and Hong Wang^*^*

**Experiments**

**Materials**: Poly(styrene-co-maleic anhydride) copolymer (SMA) was provided from Shenzhen Chemical Reagent Co. Ltd (Shenzhen, China) (Mw ~140 000, the composition ratio of maleic anhydride is 18%). 4,4-diaminodiphenylmethane (MDA) and dimethylformamide (DMF) were purchased from Aladdin Chemical Reagent (Shanghai, China). Biaxially oriented polypropylene (BOPP) film was provided by PolyK technologies. Styrene-acrylonitrile copolymer (SAN) was provided by Sigma-Aldrich. Acrylonitrile-butadiene-styrene copolymer (ABS) was purchased through Macklin Company. All chemicals were used without further purification.

**Film preparation of crosslinked polymers**: 0.5 g SAN were dissolved into 2.5 g DMF and stirred for 6 h at room temperature to obtain SAN solution, and then cast on a cleaned glass and dried in a vacuum oven. The SAN film was annealed at 80 °C for 2 h and 120 °C for 6 h, and then gradually cooled down to room temperature. For crosslinked polymers (CSMA), 0.27 mmol of MDA was added into the SMA solution (0.5 g SMA dissolved in 2.5 g DMF) and stirred for 2 h, followed by solvent-casting on a cleaned glass. Polymer blends are synthetized by tuning the composition of SAN and CSMA and following the same routines. According the CSMA contents, like 20%, 35%, 50%, 65%, 80%, labelled as SC20, SC35, SC50, SC65, SC80, respectively. Having dried in a vacuum oven, all films were obtained and peeled off from the glass. CSMA and polymer blends were heat at 155 °C oven overnight for the sufficient crosslinking reaction. The thickness of all films was in the range of 10 ± 2 µm.

**Structural characterizations**: Glass transition temperatures (*T*_g_) of polymer films were determined using a differential scanning calorimetry (DSC, TA, United States) with a heating rate of 10 K min^−1^. Surface characterization of polymers was conducted using Atomic force scanning electron microscopy (AFM, Bruke, United States) with tapping mode.

Dielectric properties and breakdown measurements: The dielectric spectra were tested in a broad temperature (30 to 160 °C) with a frequency range of 10^2^ to 10^6^ Hz using the combined setup of impedance analyzer E4980A (Keysight, United States) and DMS-2000 (Partulab, China). Breakdown strength results were measured at varying temperatures, utilizing a Trek Model 20/20C ±20 kV high voltage amplifier, applying a DC ramp voltage of 500 V s^-1^ until breakdown. The breakdown strength of polymers was measured over 12 samples.

Electric displacement–electric field (*D–E*) loop and charge-discharge measurements: The high electric field *D–E* loops of polymer films were conducted by a ferroelectric test system (PolyK, United States) with a frequency of 100 Hz. During the test, a gold electrode with a thickness of 40-50 nm and a diameter of 3 mm was sprayed on the film surface. The cycle life test uses the PK-CPR1502 charge and discharge system of the American PolyK Company. A high-voltage leakage current system (PolyK, USA), including a Keithley 6517B pA meter, a Stanford Research Systems SRS PS370 high-voltage DC power supply, and a temperature chamber, was used to record conductivity, leakage current, and thermally stimulated discharge current (TSDC).

Thermally stimulated depolarization current measurements (TSDC): The TSDC samples were sprayed a diameter of 12 mm Au electrodes on both sides. The samples were firstly polarized at 120 °C for 30 min at 20 MV m^−1^. With the polarized electric field, those samples were then rapidly cooled down from 120 °C to −50 °C (10 K min^−1^) by liquid nitrogen cooling system. The samples were maintained for 2 min at −50 °C, then heated with 3 K min^−1^ to 160 °C.

Computational details: All the DFT calculations were done within the Gaussian 09 program suite.^[1]^ The geometries were optimized at b3LYP/6-31g level. All optimizations were done without any symmetry constraints. Visualizations of molecular geometry and surface electrostatic potential were done within GaussView.

Structural optimization: Four polymer (SAN, SC35, SC65 SC80) were obtained by constructing polymers based on their molecular structure composition. Use forcite's geometry optimization module to optimize the structure of the polymer box provided by the customer, using built-in compass force field to achieve energy stable structure. The optimization of forcite itself uses the NVT ensemble. We set the iteration number to 1000 steps and the temperature to 298k until the polymer box reaches the most stable state

Molecular dynamics: Using the NPT ensemble, with a temperature of 298k, a pressure of one atmosphere, a step size of 1fs, a total simulation time of 500 ps, 100000 steps, and output results every 500 steps. Nose is used as the temperature control function, and the cutoff radius is 17A.

For four polymers in a stable state, the CN groups of the same molecular chain were extracted, and the conformers module was used to statistically calculate the torsion angles and dipole moment values of the CN groups. Universal force field was used, and the charge distribution was calculated using Qeq method. The van der Waals force cutoff radius was 17A, and the dipole moment and energy changes of the groups were recorded per 10 degrees during the torsion process.


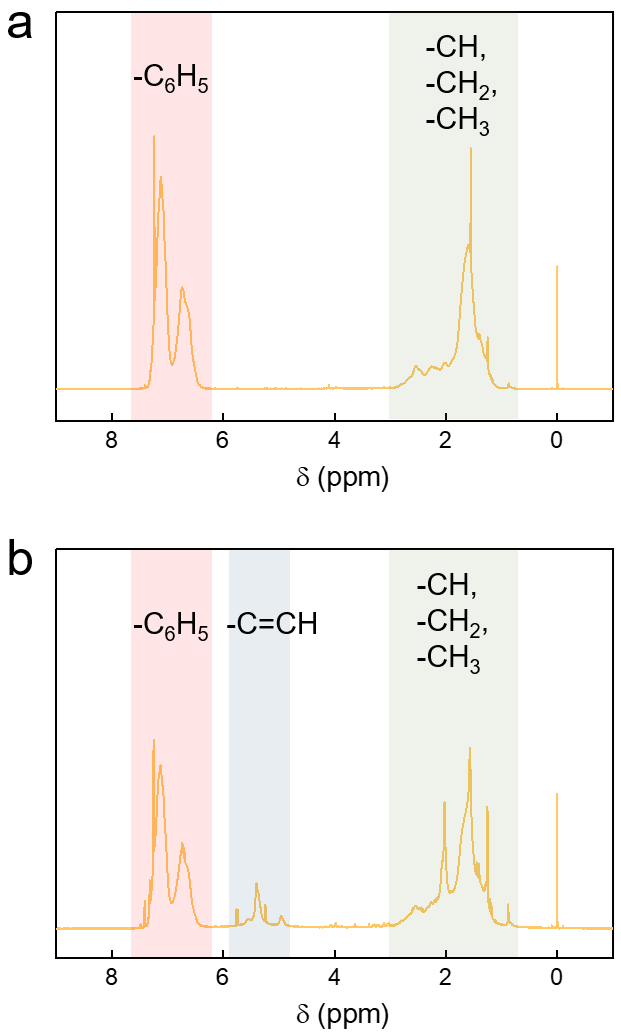


Figure S1. Molecular formula of materials for structural design.

For SAN and ABS, the signals at 6.3–7.5 ppm are assigned to the protons on the benzene ring with a relative integral area of -C_6_H_5_. The signal at 1.0-2.8 ppm can be assigned to the methyl (-CH_3_), -CH_2_ and -CH of the main chain. The signal at 5.0–5.8 ppm can be assigned to -C=CH of the ABS main chain. Therefore, the chemical composition of the copolymers is calculated by their relative integral area. The chemical composition of the SAN copolymer was calculated from ^1^HNMR based on the integral of peaks at 6.3 ppm to 7.5 ppm and 1 ppm to 2.8 ppm, leading to the result that the copolymer contains 38.6 mol.% acrylonitrile and 61.4 mol.% styrene units. The chemical composition of the ABS copolymer was calculated based on the integral of peaks at 6.3 ppm to 7.5 ppm, 5.0 ppm to 5.8 ppm and 1 ppm to 2.8 ppm, leading to the result that the ABS copolymer contains 35.3 mol.% acrylonitrile, 17.5 mol.% butadiene, and 47.2 mol.% styrene units.


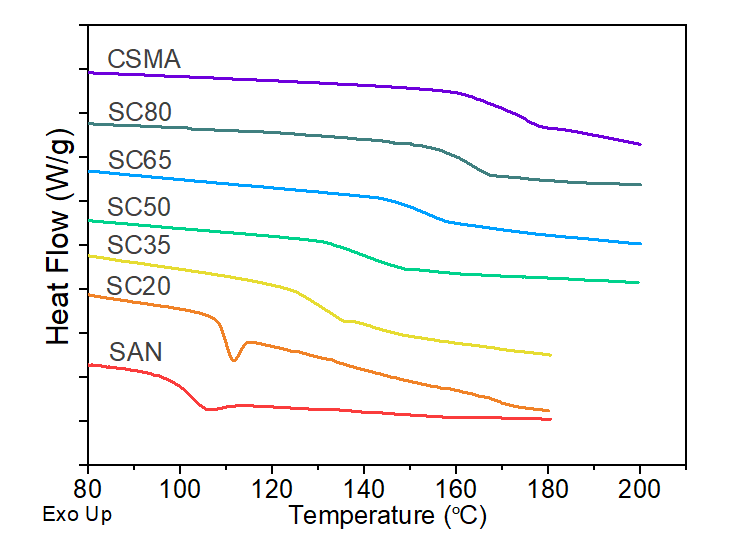


Figure S2. Investigation of polymers and blends by the differential scanning calorimetry (DSC) method.


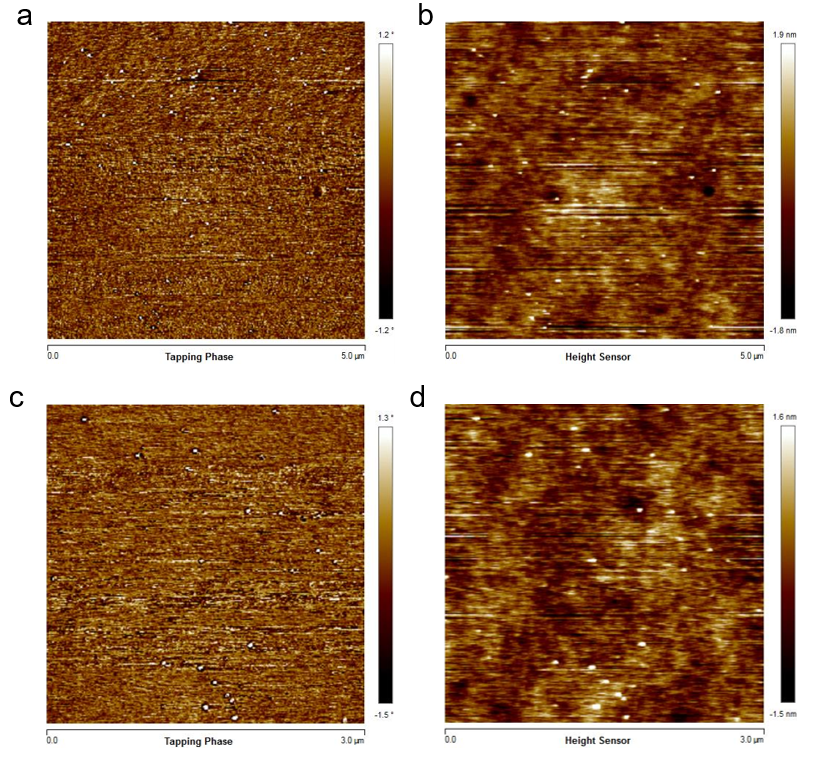


Figure S3. AFM scan results of (a and b) SAN and (c and d) CSMA.


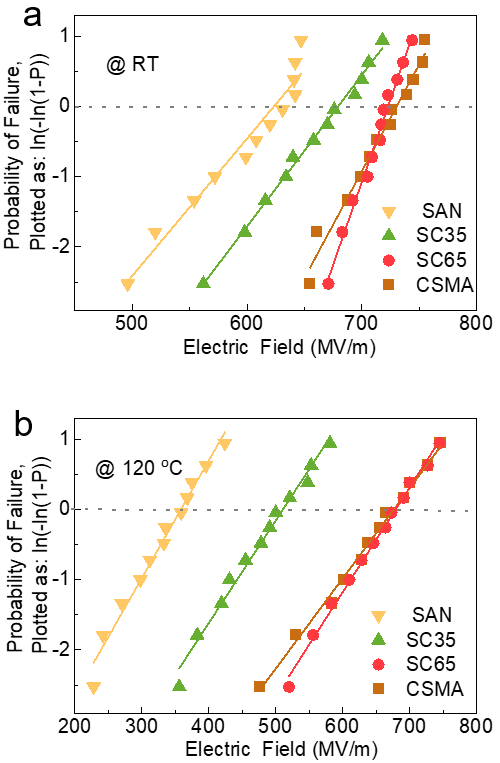


Figure S4. The breakdown Weibull distribution of SAN, CSMA, and SC blends at (a) room temperature and (b) 120 ^o^C.

Figure S5. The enhancing the modulus of the SC blend materials as compared to SAN polymer at 120 ^o^C.

The higher modulus of SC65 compared to SAN is due to the rigid main chain and cross-linked structure of CSMA, resulting in a higher glass transition temperature and higher mechanical strength. Therefore, although the chain is fully extended, the increase in modulus of SC65 mainly contributed to the rigid crosslinked structure.


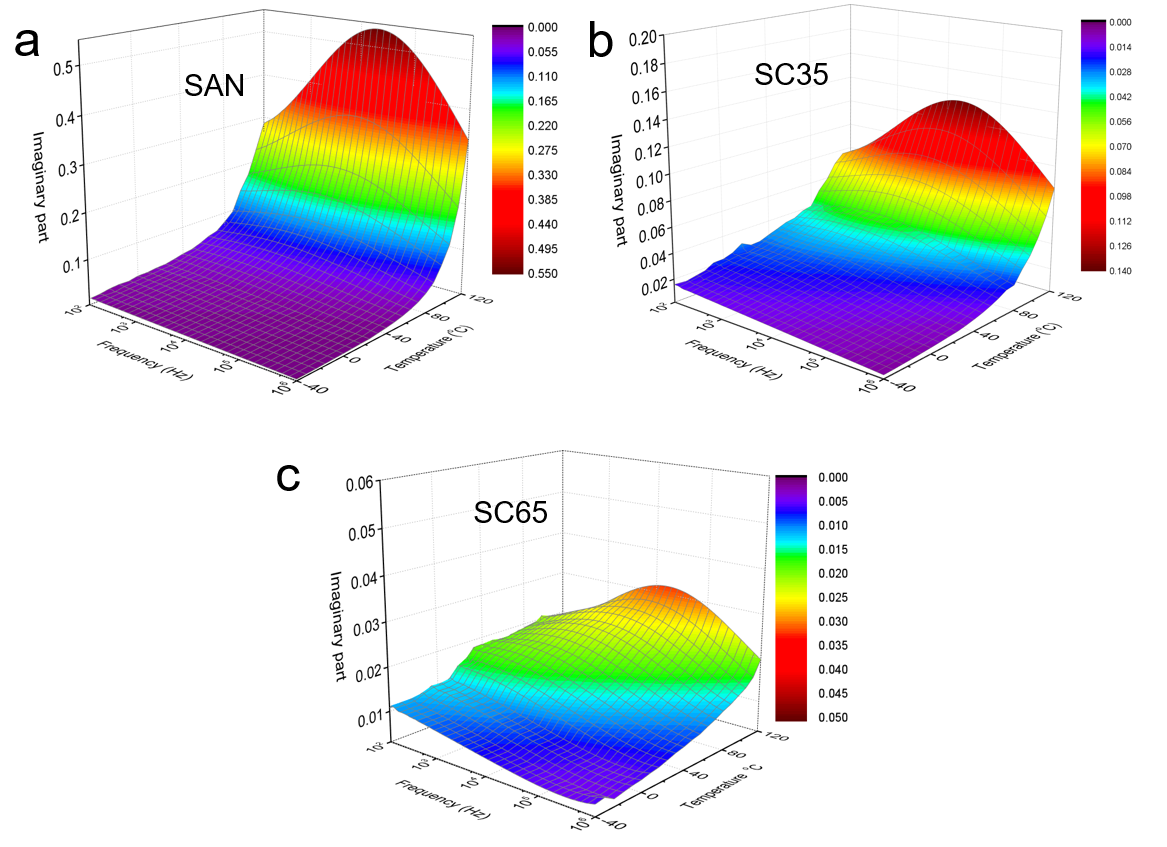


Figure S6. The three-dimensional plot of the imaginary part of dielectric response as a function of the temperatures and frequencies. (a) SAN, (b) SC35, and (c) SC65.


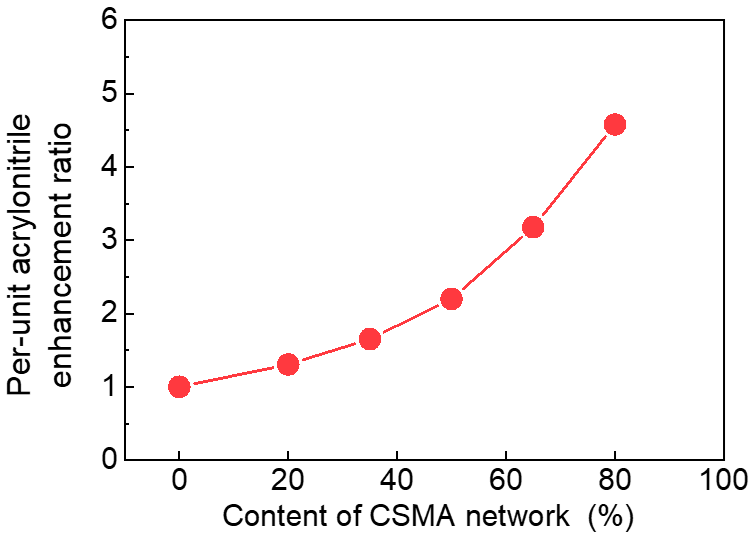


Figure S7. The per-unit acrylonitrile enhancement ratio of dielectric constant in SAN and SC blends.


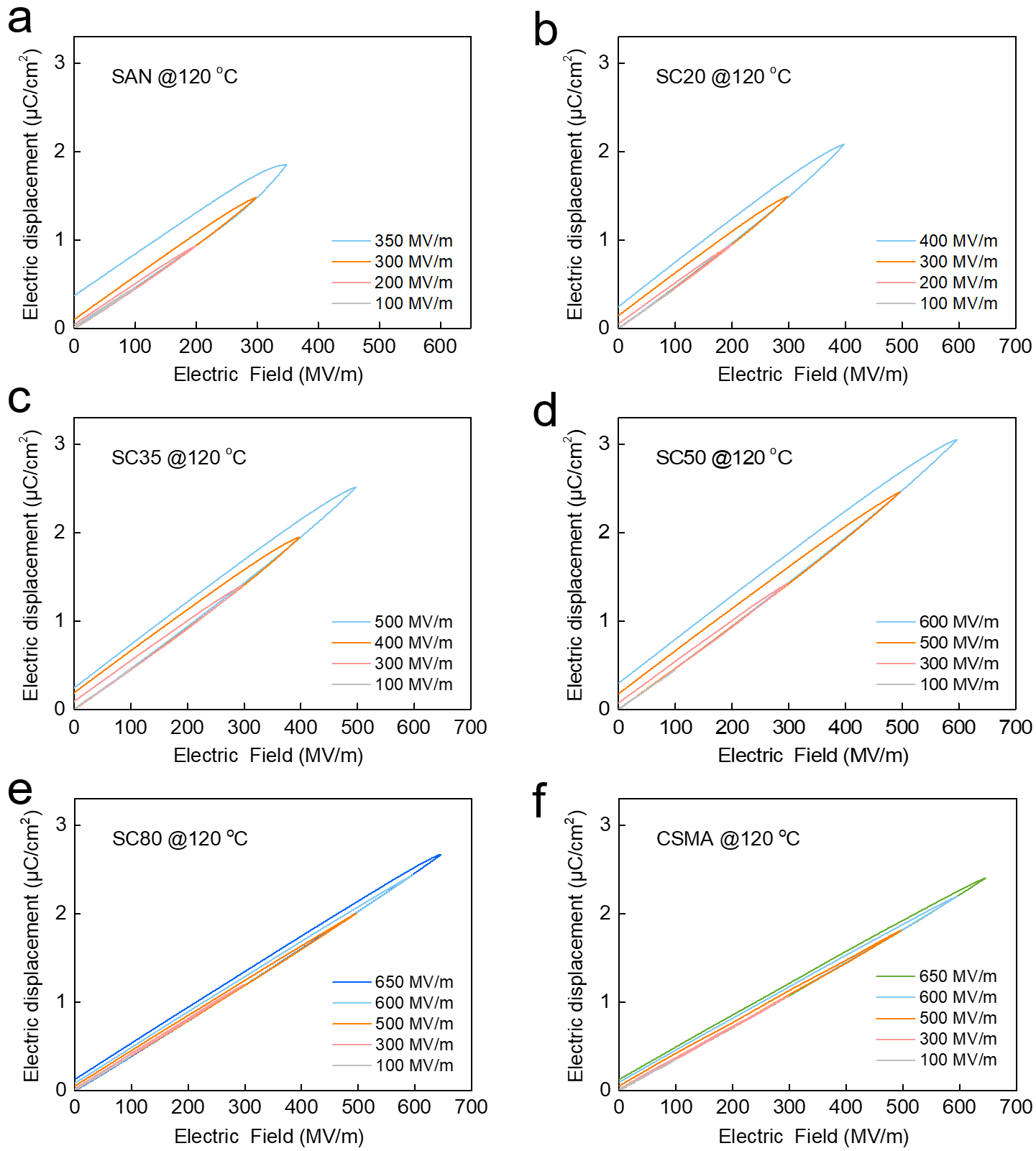


Figure S8. *D-E* loops of polymers and blends films measured at 120 °C.


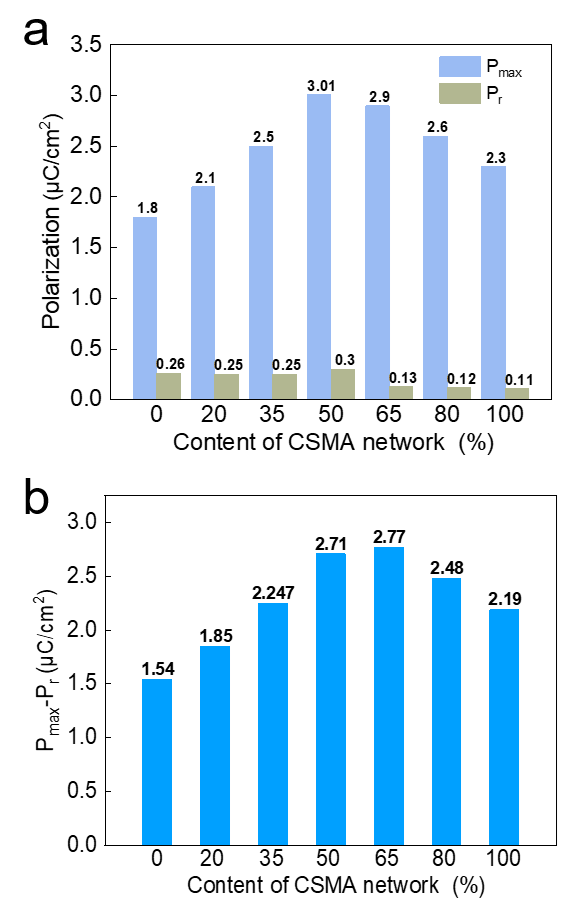


Figure S9. (a) Maximum polarization and (b) remanent polarization of polymers and blends films measured at the maximum electric field and 120 °C.


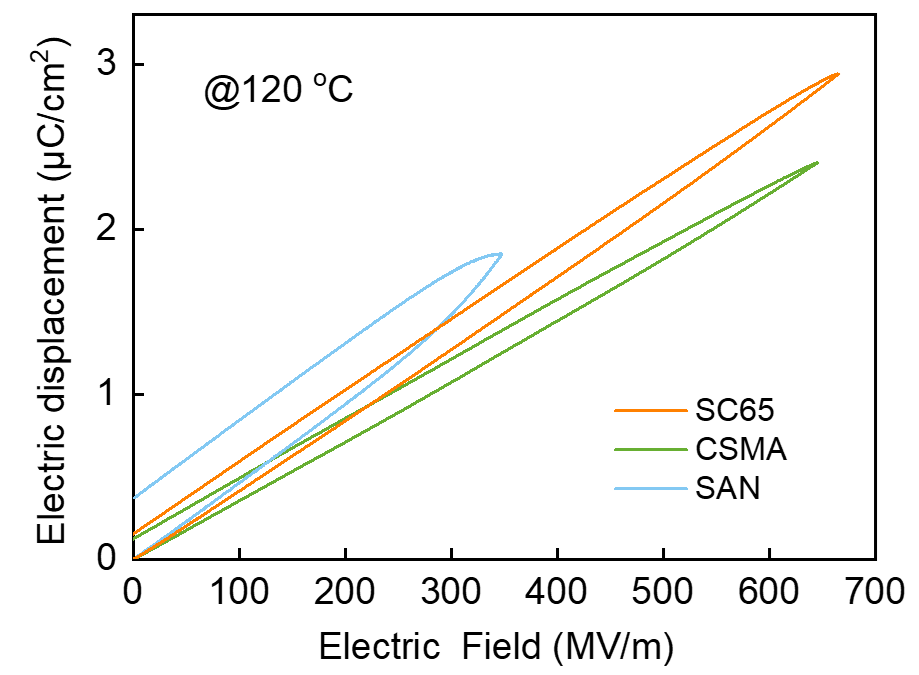


Figure S10. *D-E* loops of polymers and SC65 blend films measured at 120 °C.


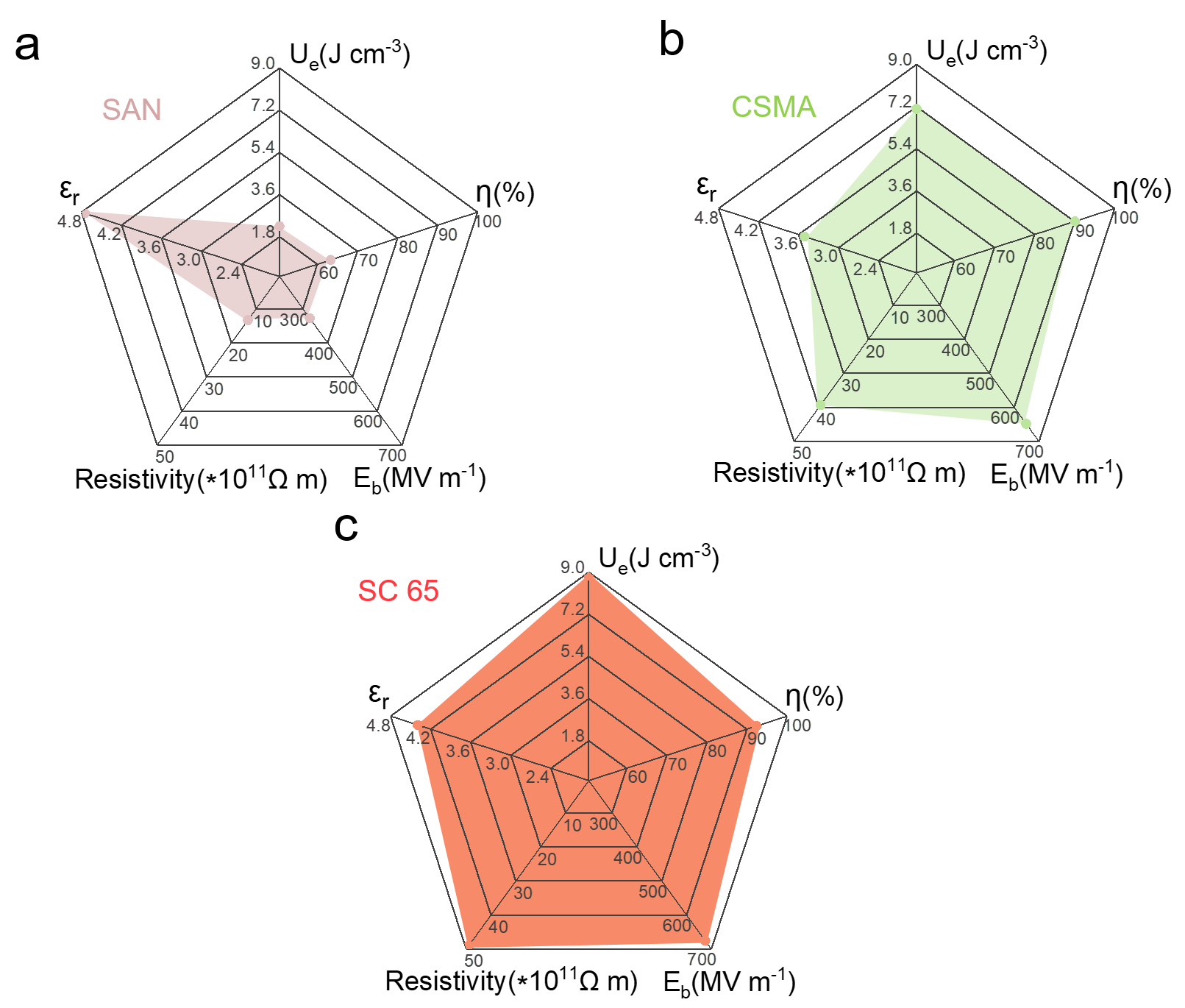


Figure S11. Radar map of comprehensive dielectric performances at 120 °C.


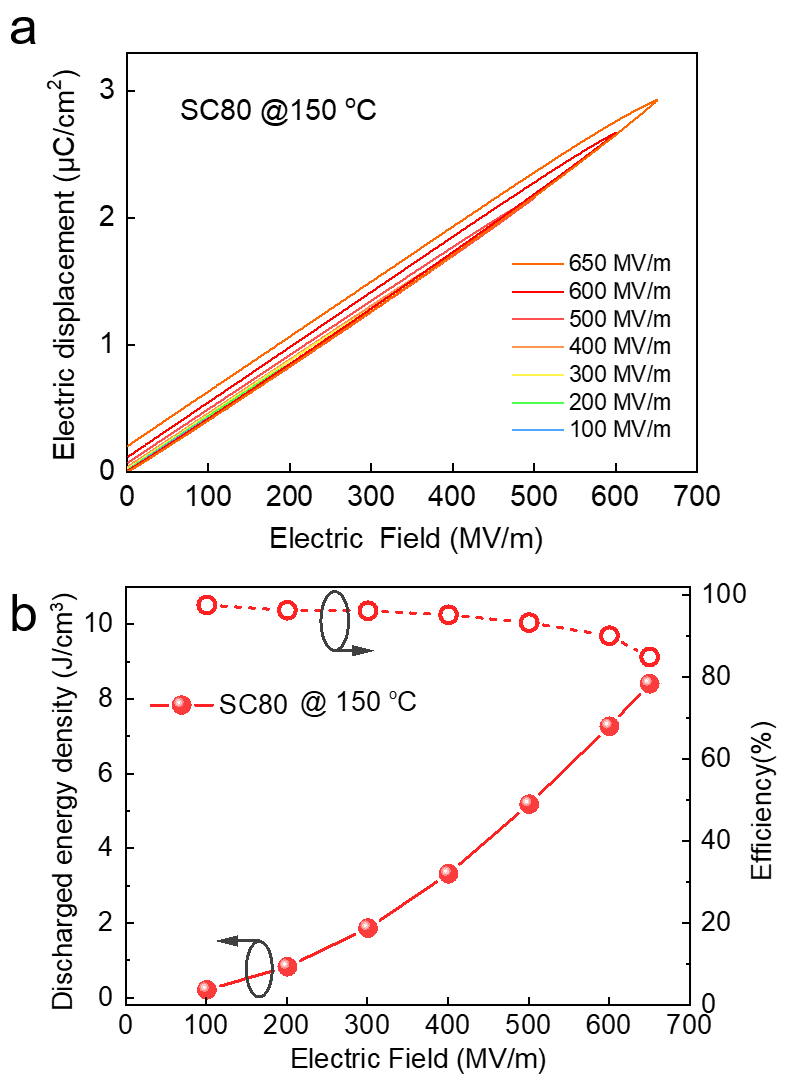


Figure S12. (a) *D-E* loops of SC80 film at 150 °C. (b) The discharged energy density and efficiency *vs*. electric field of the SC80 at 150 °C.


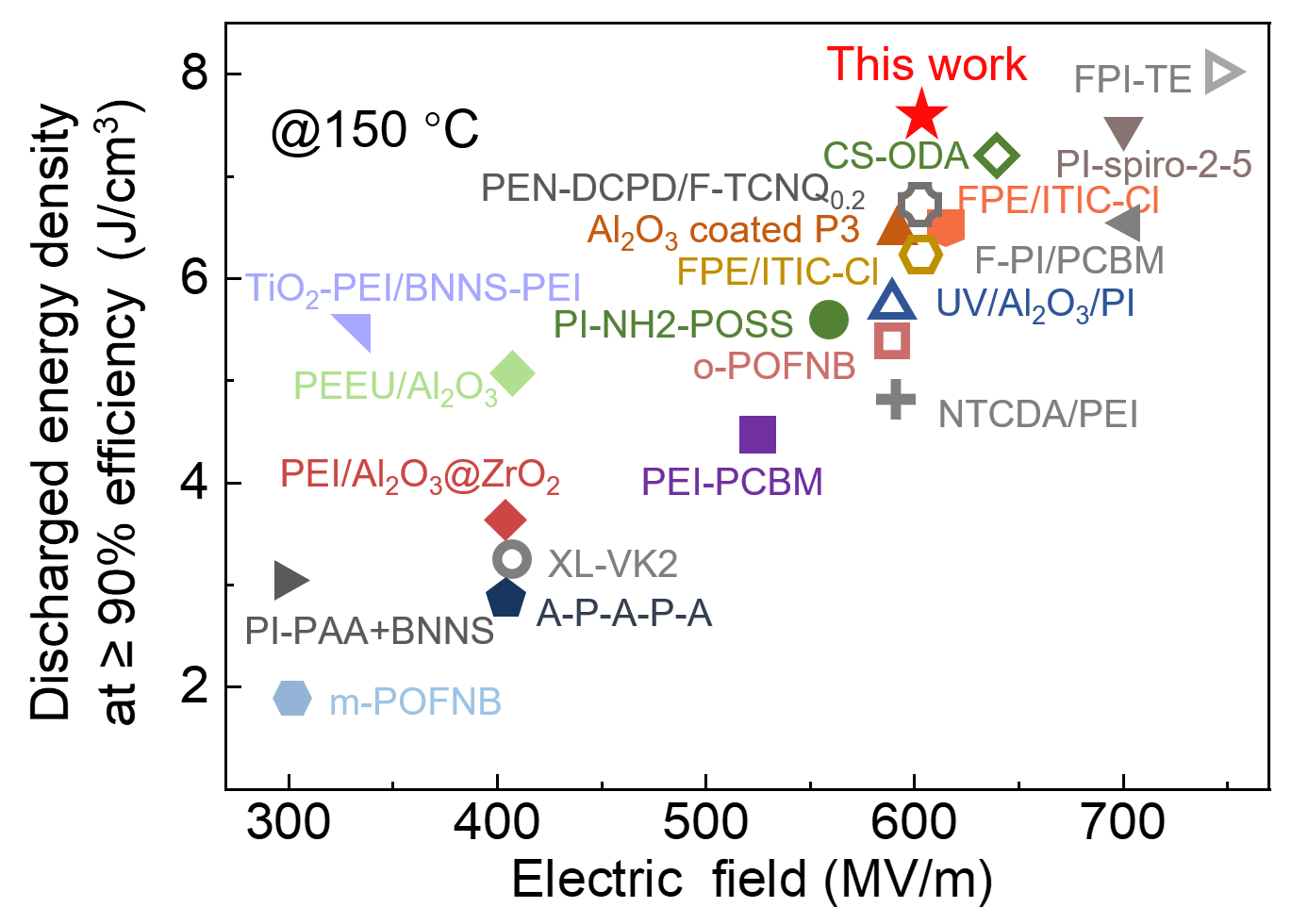


Figure S13. Maximum polarization and remanent polarization of CSMA and SC80 films measured at the electric field of 600 MV m^-1^ and 150 °C.


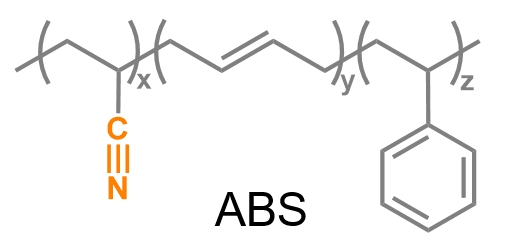


Figure S14. ABS molecular structure.


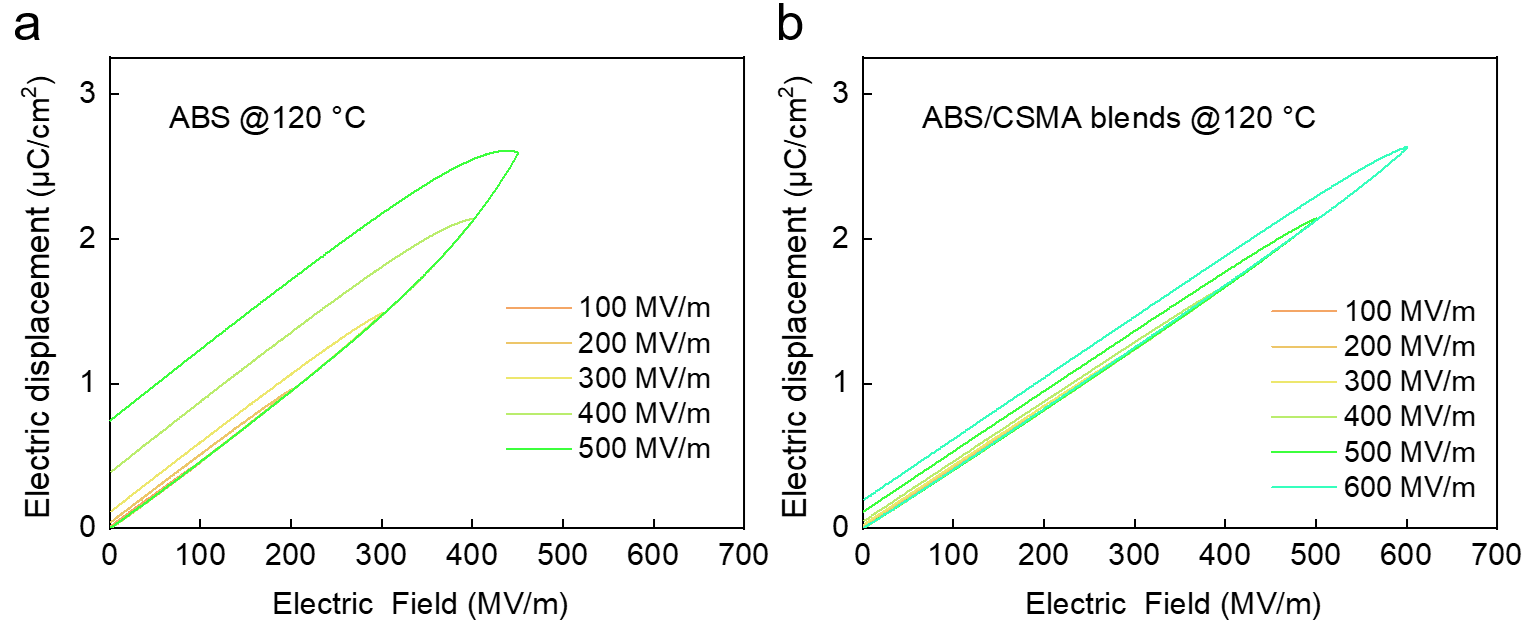


Figure S15. (a) *D-E* loops of ABS film at 120 °C. (b) The discharged energy density and efficiency *vs*. electric field of ABS/CSMA at 120 °C.


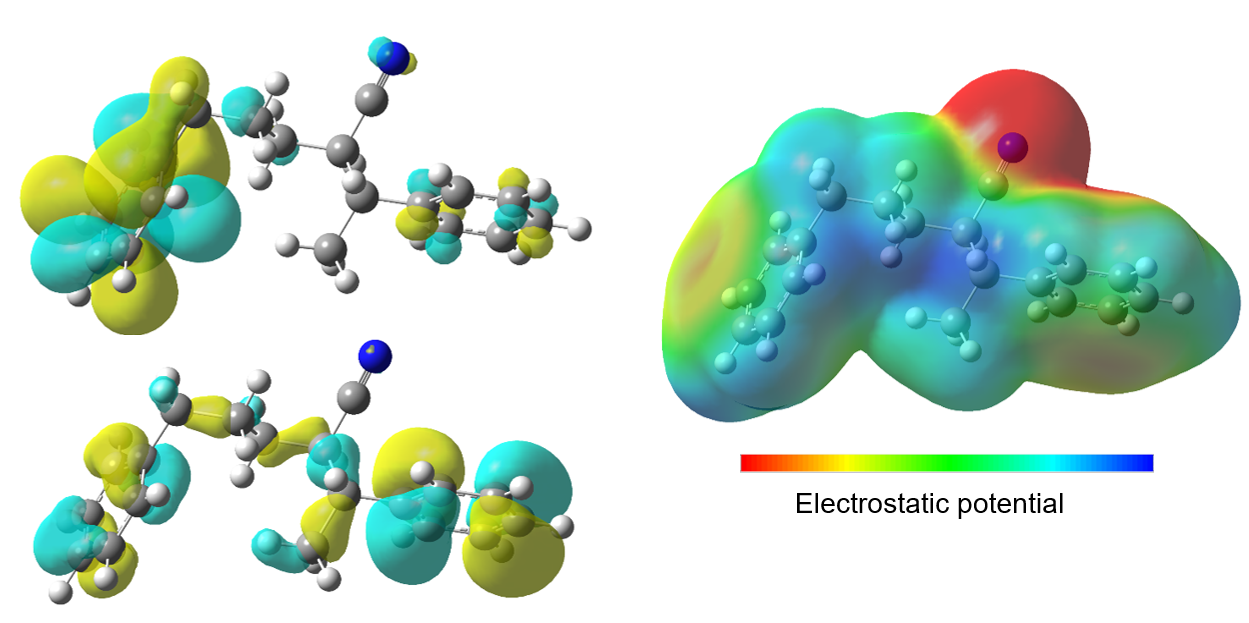


Figure S16. Backbone structures and electrostatic potential distribution of SAN.


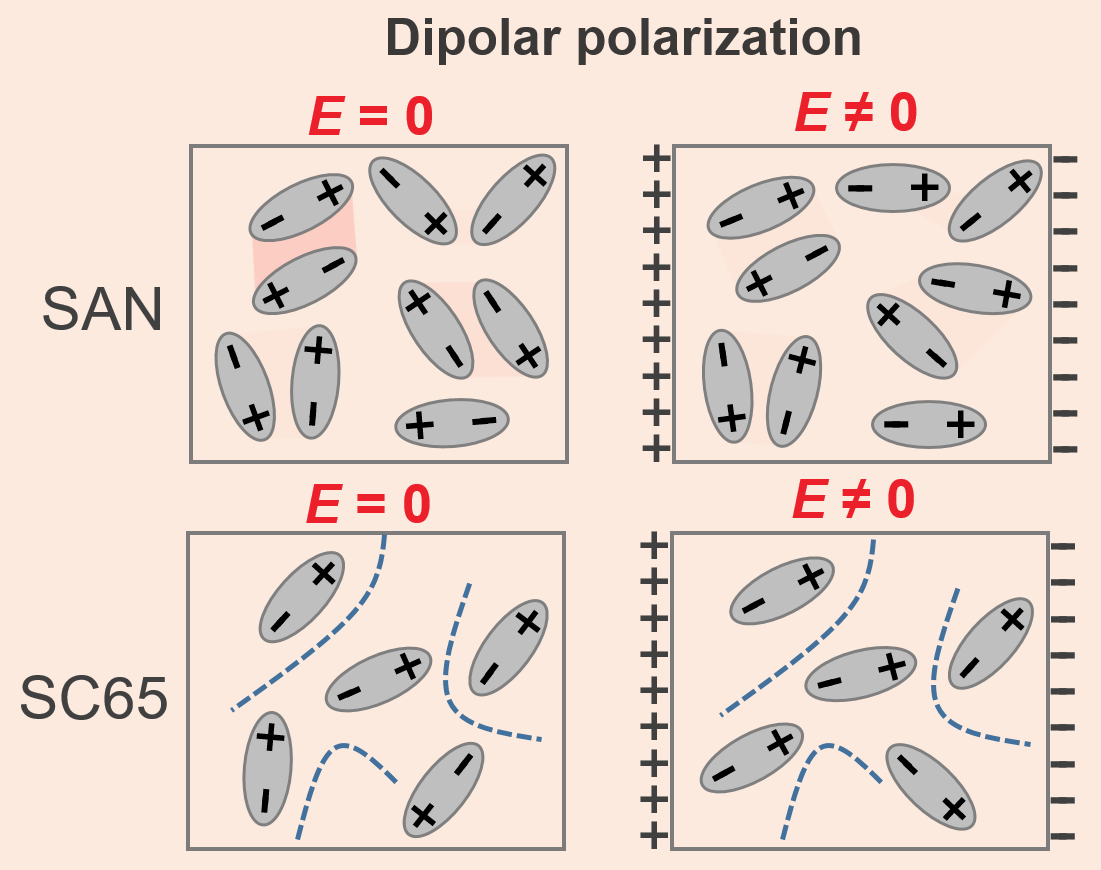


Figure S17. Schematic diagram of electric field induced orientation.


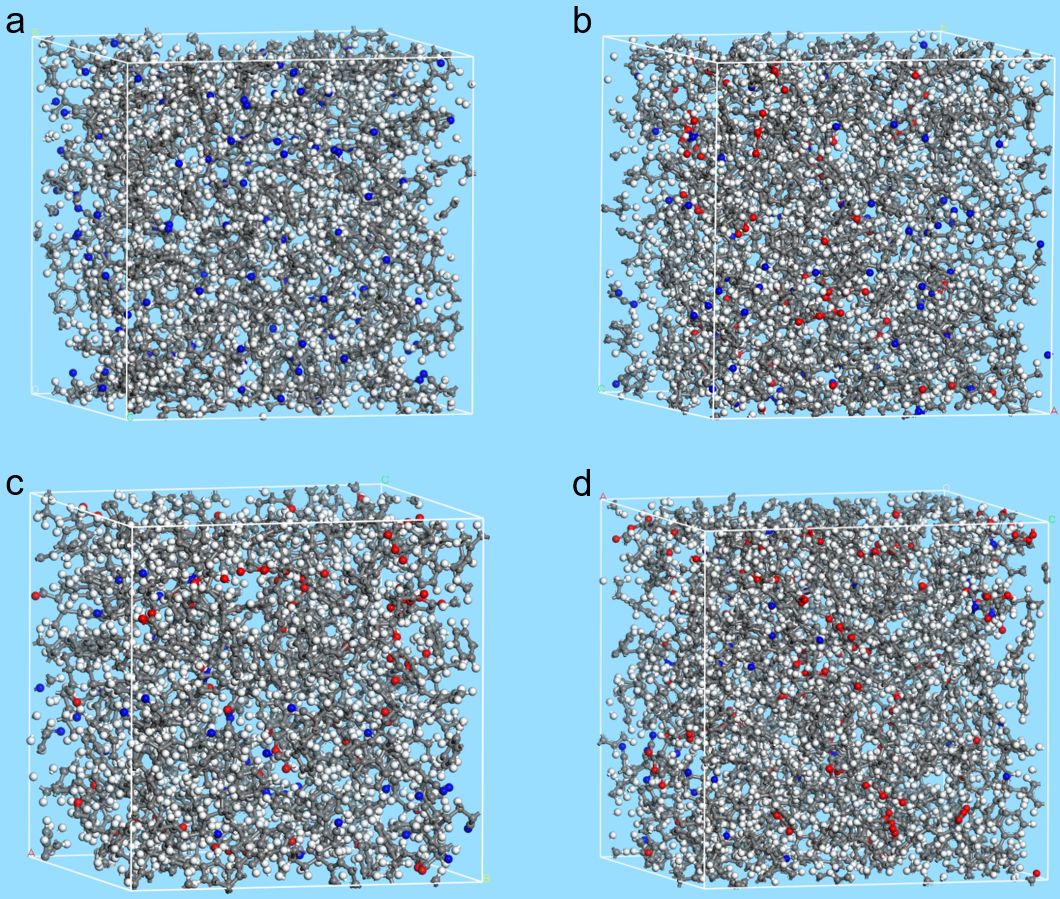


Figure S18. The optimized structures of (a) SAN, (b) SC35, (c) SC65, and (d) SC80 by molecular dynamics simulations.


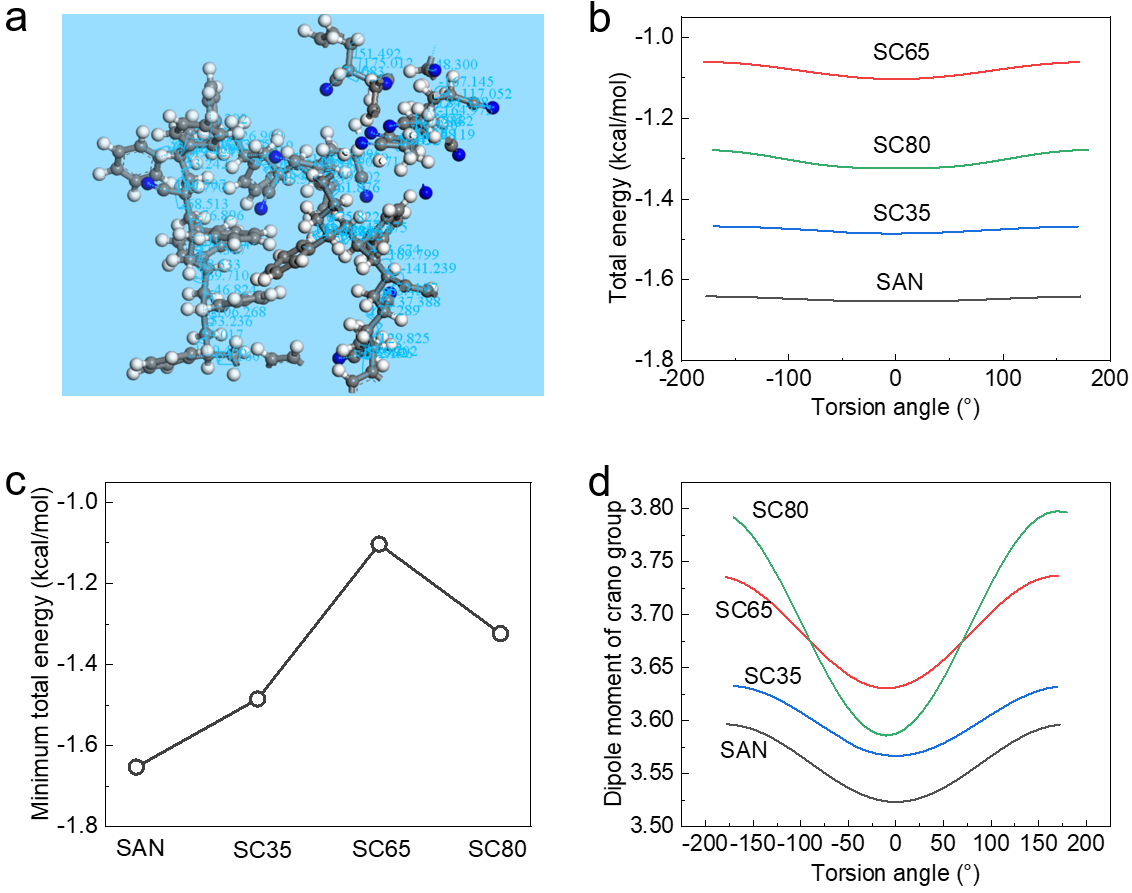


Figure S19. (a) The selected segment of optimized structures for analysis of torsion angle of cyano group. (b) Total energy variation of cyano group with torsion angle in SAN and SC blends. (c) Minimum total energy of cyano group in SAN and SC blends. (d) Dipole moment of cyano group with torsion angle in SAN and SC blends.

We observed the relationship between the dipole moment and torsion angle of cyano groups in SAN and SC blends. The dipole moment of the cyano group also varies slightly in different polymers, gradually increasing in SAN, SC35, and SC65, indicating that the contribution of a single cyano group to the overall polarity is increasing. In stable state (0° torsion angle), the contribution of the cyano dipole moment of SC80 is slightly lower than that of SC65. Therefore, we further evaluated experimental data related to dielectric constant by molecular dynamics simulations, and analyzed the internal -CN dipole contribution of the polymers at the molecular level.


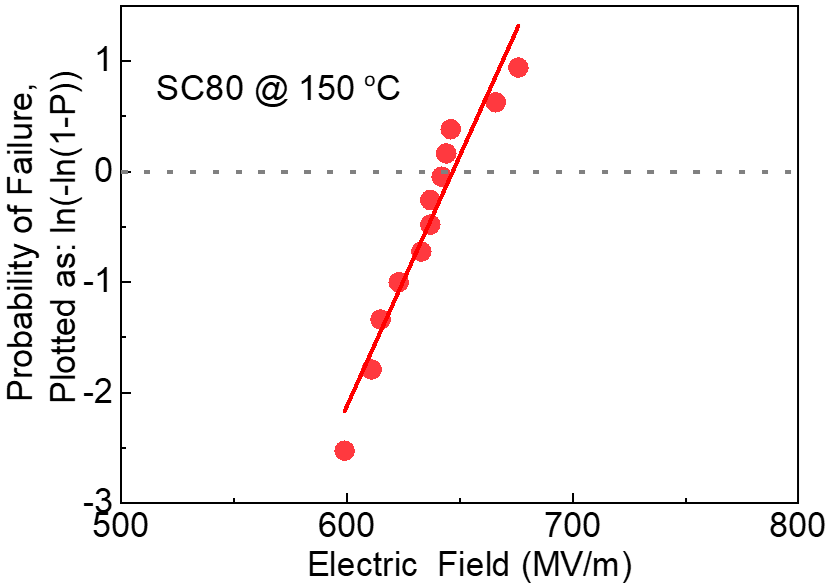


Figure S20. (a) The Weibull breakdown strength of SC80 thin film at 150 °C.


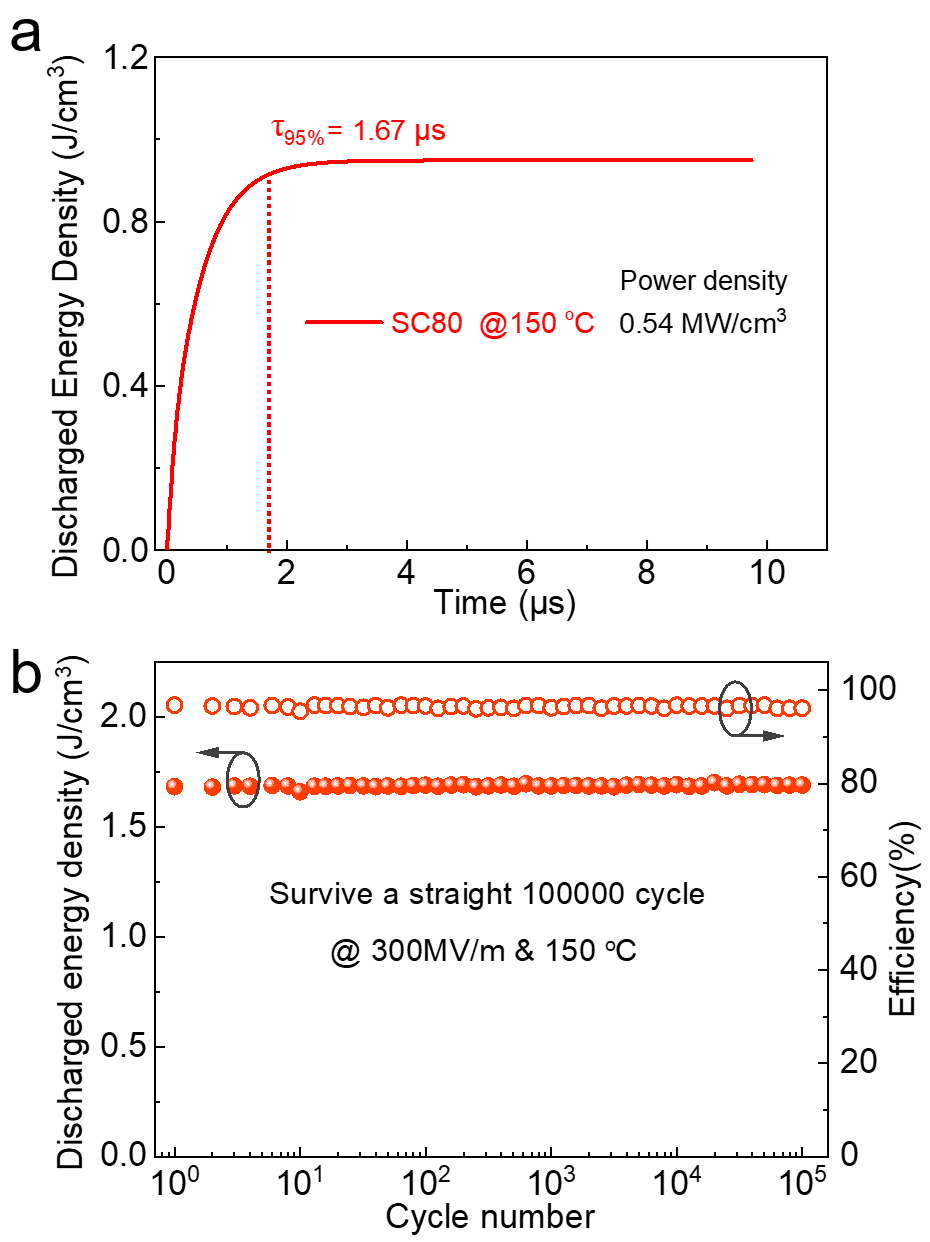


Figure S21. (a) The discharged energy density as a function of time of SC80 film at 150 °C. (b) The cycling performance of SC80 film at 150 °C.


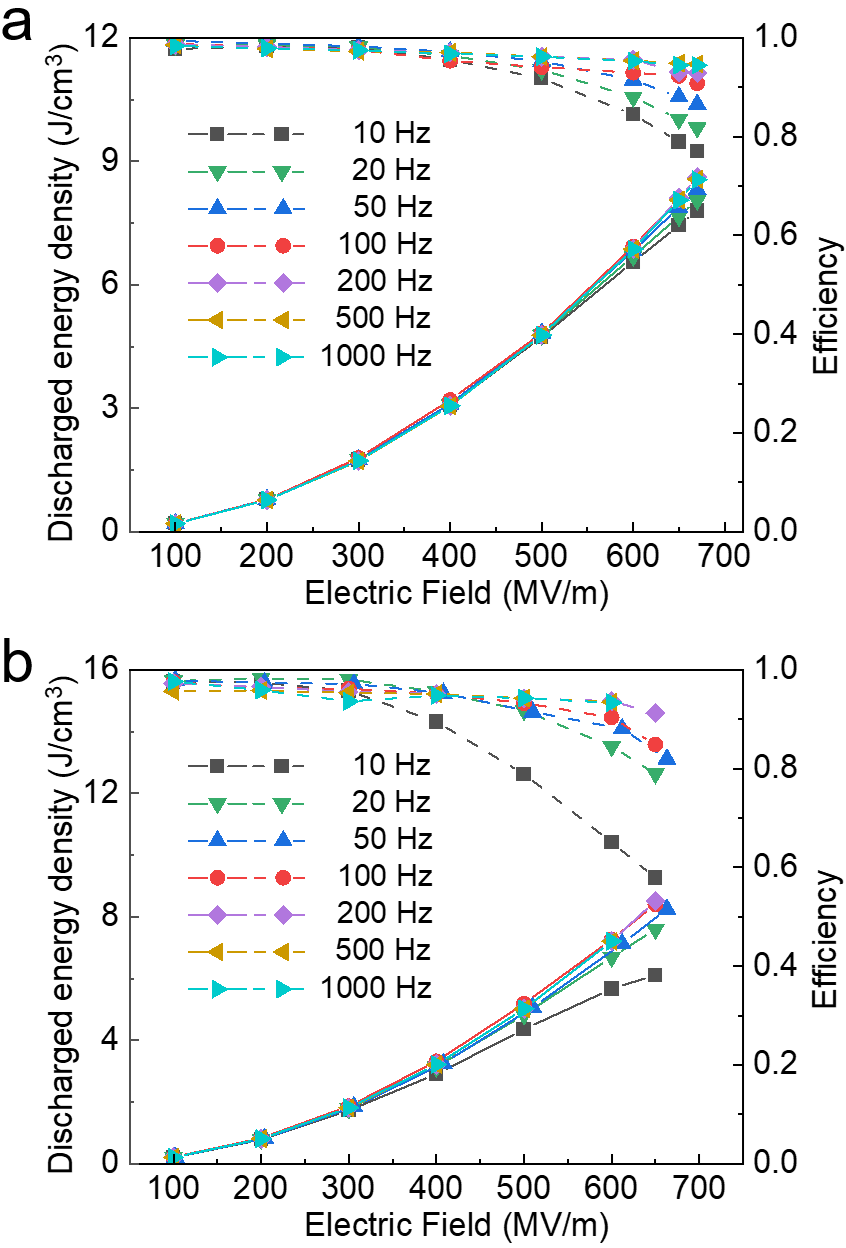


Figure S22. The capacitive performance of (a) SC65 (@120°C) and (b) SC80 (@150°C) from 10 Hz to 1000 Hz.
